# Proton Beam Therapy and the AYA Sarcoma Patient Journey: Highlighting Needs from Diagnosis to Survivorship

**DOI:** 10.3390/cancers17213402

**Published:** 2025-10-22

**Authors:** Margaret M. Harris, Safia K. Ahmed

**Affiliations:** Radiation Oncology Department, Mayo Clinic, East Mayo Boulevard, Phoenix, AZ 85054, USA

**Keywords:** adolescent and young adult, proton beam therapy, sarcoma, developmental milestones, fertility, sexual health, financial burden, palliative care, survivorship, social media, future direction

## Abstract

**Simple Summary:**

A sarcoma diagnosis is devastating at any period in life but can be particularly disruptive to those who are still in the stages of development. An adolescent and young adult (AYA) patient has been defined as a patient aged 15–39 at the time of diagnosis. Research has proven this patient population to be unique through the exploration of challenges and needs that positively impact outcomes. Proton beam therapy (PBT) is a component of cancer care that has been found to be underutilized in the AYA patient population. This review will highlight research that has been completed that supports the use of PBT, present the many challenges that this unique patient population faces, discuss interventions that have been utilized, and present opportunities for growth to better serve an AYA sarcoma patient.

**Abstract:**

**Background**: Adolescents and young adults (15–39 years of age at time of diagnosis: AYA) with sarcoma are a unique patient population. The objective of this review is to examine the literature outlining the benefits of proton beam therapy (PBT) for treatment of AYA sarcoma patients, barriers to PBT, evaluation of AYA-specific considerations and challenges, and exploration of future opportunities for improvements in care. **Methods**: An electronic search was conducted using databases and online search engines, primarily PubMed. The search criteria included studies and reviews completed from 2015 to 2025. **Results:** 57 articles were reviewed and categorized into sections: PBT for the treatment of the AYA patient, barriers to PBT, AYA-specific considerations and challenges, and future directions for the care of an AYA patient. **Conclusions**: Through this review, PBT can be deemed necessary when treating AYA sarcoma patients with radiation therapy to decrease long-term therapy-related toxicities. Furthermore, considerations for caring for an AYA sarcoma patient must extend beyond evidence-based treatment plans and must embrace the patient as a whole person through acknowledgement of the challenging impact on physical, mental, and social well-being from symptoms to diagnosis, diagnosis to treatment, and treatment to survivorship.

## 1. Introduction

Each year, there are approximately 90,000 new cases of cancer in adolescents and young adults (AYAs: 15–39 years of age at diagnosis) [1], comprising up to 4% of all new cancer diagnoses in the US. In 2022, an estimated 377,621 cancer-related deaths occurred among AYAs worldwide [2]. Sarcomas account for almost 11% of all cancers in the AYA population [3]. Sarcoma is defined as a broad group of cancers that start in the bones and the soft tissues, which include muscle, fat, blood vessels, nerves, tendons, and the lining of the joints, with more than 100 different entities described in the World Health Organization 2020 classification [4,5]. Multimodal treatments such as surgery, chemotherapy, and radiation are utilized in the treatment of sarcomas. The multimodal approach improves the likelihood of a cure and decreases the risk of recurrence. To achieve a multimodal treatment plan for a patient, a multidisciplinary approach is essential. This involves a team of sarcoma experts: radiology, pathology, surgical and orthopedic oncology, radiation oncology, and medical oncology. Through collaboration among healthcare professionals, optimal treatment plans with guidance from the National Comprehensive Cancer Network (NCCN) can be developed for patients. Radiation therapy is an integral part of treatment for AYA patients, particularly those with a sarcoma diagnosis. While radiation therapy is mainly utilized preoperatively to increase local control in resectable tumors, there is also a role for radiation in the treatment of non-resectable primary tumors, in oligometastatic situations, or for palliative purposes [6]. Like pediatric patients, AYA patients are at an increased risk of developing long-term radiation toxicities. Toxicities may include organ dysfunction, hormone deficiency, fertility difficulties, cardiovascular disease, and secondary malignancies [6]. Therefore, it is imperative to review the importance of proton beam therapy (PBT) for the treatment of the AYA patient. In addition to treatment-related impact, a sarcoma diagnosis as an AYA can be particularly disruptive as it occurs in a developmental stage in life. A diagnosis can cause physical, mental, social, and financial implications during treatment, and as patients transition to survivorship. This review will serve to highlight opportunities for the development of interventions and strategies to better support an AYA patient throughout the continuum of care, specifically at radiation oncology clinics. This will be addressed through exploring the necessity of PBT for the treatment of an AYA sarcoma patient, outlining the challenges an AYA patient faces, discussing tools to support an AYA patient, and exploring opportunities for growth to better support this unique patient population. The intention of this review is for radiation oncology teams to have an understanding of the unique challenges an AYA patient faces, and to help facilitate radiation oncology teams to serve as advocates for these patients through sarcoma treatment.

## 2. Materials and Methods

An integrative literature review was to accentuate the necessity of PBT for the treatment of an AYA sarcoma patient, explore the challenges that the AYA sarcoma population face, identify tools to support an AYA patient, and present future opportunities to improve care An electronic search was conducted using databases and online search engines, primarily PubMed. A combination of keywords was utilized: adolescent and young adult; proton beam therapy; sarcoma; developmental milestones; fertility; sexual health; financial burden; palliative care; survivorship; social media; and future direction. Search criteria included studies and reviews completed from 2015 to 2025. A final set of 57 references were utilized for this review.

## 3. Results

### 3.1. Diagnosis and Access to Expert Care

Advocacy for the AYA sarcoma patient begins at the time of diagnosis. Sarcomas account for more than 1% of all incidences of care, outlining the rarity of this cancer [7]. Due to the rarity of sarcomas, expedited referral to institutions with sarcoma experts is essential. Moreover, research has demonstrated that rapid referral to an expert sarcoma center improves patient outcomes [8]. The United Kingdom established a guideline to assist providers to more appropriately identify patients that would benefit from a referral to a sarcoma expert. Referral is recommended in patients that have a soft tissue mass greater than 5 cm, a painful lump, a lump that is increasing in size, a lump of any size that is deep to the muscle facia, or recurrence of a lump after a previous excision [9]. Utilization of a set guideline may prove beneficial for providers with limited or no experience with sarcoma, so that patients may be appropriately referred. Soft tissue sarcomas are often diagnosed unexpectedly after surgery and therefore, surgical excision may be incomplete. A retrospective chart review. investigated the reliability of primary histopathological assessments, whether revisional surgery improved resection status, and the prognostic value of the residual tumor at re-excision. This chart review also explored patients who had undergone the primary excision in a non-comprehensive cancer center (CCC). Results of the study revealed that 53% were referred to a CCC after initiating therapy elsewhere, presentation status was established with certainty in 96 cases (87.3%), a “whoops’ procedure was performed in 71 cases (74%), recurrent local disease, either after a single excision or repeated excision, was found in 22 cases (22.9%), and just three cases (3.1%) presented after incisional biopsy [10]. The reviewed guidelines and the chart review provide evidence for referral to CCCs and impact on the outcome, as well as the necessity of expertise to properly diagnose and effectively treat sarcoma patients.

A 2015 study survey assessed primary care practitioners (PCP) in Minnesota on their familiarity with sarcoma guidelines and confidence in managing sarcoma cases. A total of 80 physicians and 32 nurse practitioners completed the survey, which included questions regarding clinical practice, training and education on sarcoma, familiarity with the sarcoma guidelines, case presentations, and diagnosis delays in sarcoma patients. Results revealed low familiarity and confidence among both groups, with only 24 of 74 physicians (32%) and 19 of 29 NPs (66%) never having encountered a case of soft tissue sarcoma [9]. Reported contributing factors to delays in diagnosis included patient delays in seeking care, clinical time constraints, insurance barriers, delays in imaging, and misinterpretation of imaging results [9]. Results of the survey echo the existing literature on the rarity of sarcoma and highlight the necessity of education on sarcoma for general practitioners to reduce delays in diagnosis and improve sarcoma patient outcomes.

Rapid referral to expert sarcoma centers has been found to positively impact patient outcomes. To ensure adequate care and promote improved outcomes, a team in France developed a mobile app (Sar’Connect) based on an algorithm designed by radiologists from the French Sarcoma Group. Sar’Connect provides personalized advice for the management of patients and contact information for the closest sarcoma expert center. A retrospective study was completed to assess the app’s potential benefit in reducing the time interval for a patient referral to an expert center. Results revealed a potential benefit of more than a 7-month reduction when referring to sarcoma expert centers using the designed mobile app [11], shedding light on the opportunity to leverage digital health to improve sarcoma patient outcomes.

With growing attention on the importance of seeking care from sarcoma specialists to improve patient outcomes, a multidisciplinary group led by the Sarcoma Patient Advocacy Global Network (SPAGN) defined and developed criteria to describe a sarcoma specialist facility. The goal of these efforts was to provide guidance and support to patients and families in navigating access to sarcoma specialist care. Some of the identified principles include a multi-disciplinary team of experts that discuss patient care throughout the continuum of care, availability of MRI, experienced pathologists that review the primary tissue sample or provide a second opinion, a caseload of 100+ sarcoma patients each year, involvement of the facility with regional or national sarcoma specialist groups, access to new techniques that aid in the care of sarcoma patients, and the opportunity for patients to participate in clinical trials [12]. The criteria discussed outlines the many components that characterize a sarcoma specialty center. Resources such as The Sarcoma Alliance exist to assist patients and families in identifying sarcoma specialist centers [12]. The website lists sarcoma centers within the United States, as well as international sarcoma centers. Beyond sarcoma specialist care, access to facilities that offer proton beam radiation therapy has proven to be beneficial when caring for an AYA sarcoma patient.

### 3.2. Proton Beam Therapy (PBT) in AYA Patients

#### 3.2.1. Benefits to PBT

**Figure 1:**
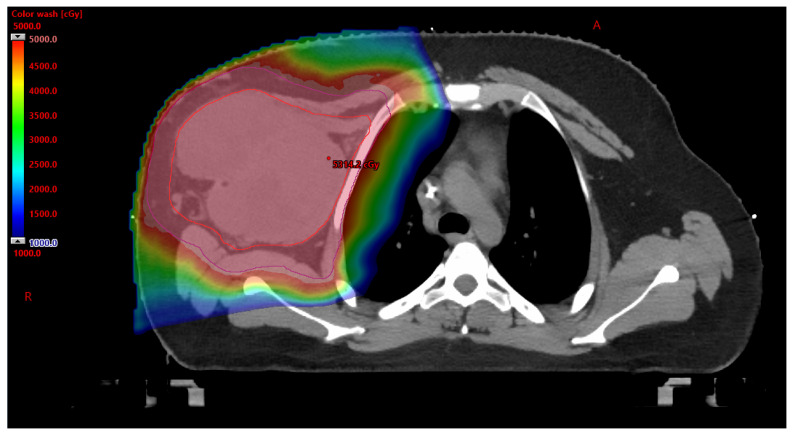
PBT utilized for treatment of the right anterior chest wall for an 18-year-old AYA patient with synovial sarcoma. Patient was seen in the sarcoma multidisciplinary clinic which determined the recommended treatment plan of neoadjuvant chemotherapy, followed by preoperative radiation therapy. A: anterior R: right.

**Figure 2:**
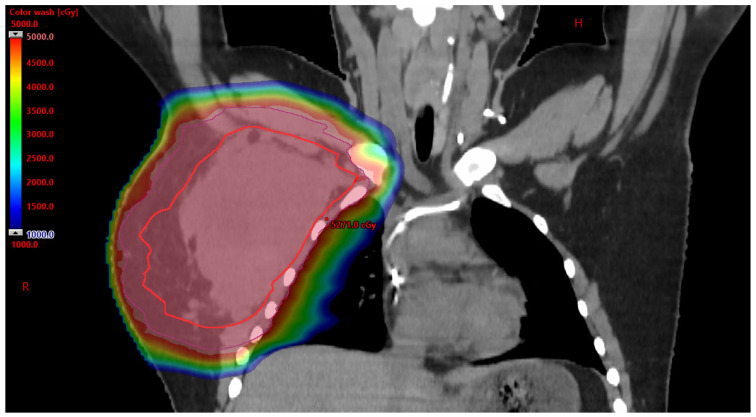
Additional view of the designed PBT treatment plan, which demonstrates PBT ability to avoid dosage to the right lung and heart. Mean heart dose was 0.15 Gy and mean right lung dose was 13.4 Gy. There was no radiation dose to the left lung. H: head R: right.

While the FDA approved PBT for the treatment of cancer in 1988, approval of PBT in the AYA population often proves to be difficult [13]. As part of an AYA sarcoma patient’s cancer care, radiation therapy is often utilized. With radiation therapy comes acute side effects, as well as long-term side effects. The AYA population is particularly susceptible to the development of radiation-therapy-related late toxicities—including organ dysfunction, hormone deficiency, fertility difficulties, cardiovascular disease, and secondary malignancies [14]. Due to young age and expected years of survivorship, limiting short-term and long-term side effects is essential for positive outcomes related to the control/cure of the disease and quality of life. Compared to photon-based radiation therapy, PBT can provide a clear dosimetric advantage by eliminating the exit dose and reducing the entry dose without compromising the tumoricidal dose, see Figure 1 and Figure 2 [14]. A clinical investigation was published in 2020 which explored treatment outcomes after PBT for Ewing sarcoma of the pelvis. The investigation explored overall survival and tumor control rates in thirty-five patients aged less than 21 with no metastatic pelvic Ewing sarcoma who received PBT and chemotherapy between 2010 and 2018. The findings highlighted the benefits of PBT. Patients with large tumors (≥8 cm) who underwent definitive PBT with a higher dose (>59.4 Gy RBE) remained free from tumor recurrence (*n* = 5) [15]. While this investigation was focused on patients under 21 years of age, it highlights the benefit of PBT providing local control and dose escalation without significant radiation toxicity for patients with Ewing sarcoma. Advantages of PBT for Ewing sarcoma of the cranium and skull base were explored through review of 25 patients ≤ 21 years old with non-metastatic Ewing sarcoma of the cranium and skull base that were treated between 2008 and 2019. Results found that the four-year local control, disease-free survival, and overall survival rates were 96%, 86%, and 92% [16], with none of the studied patients having developed a secondary malignancy. These findings call attention to the benefits of PBT in the treatment of AYA sarcoma patients and the opportunity to explore the role of PBT in the care of all AYA cancer patients. Despite the proven benefit of PBT in the treatment of AYA sarcoma patients, barriers to use have been identified.

#### 3.2.2. Barriers to PBT

Minimizing long-term radiation toxicity is a key priority for AYA sarcoma patients, particularly as 86% of AYAs diagnosed with cancer survive at least five years after diagnosis [17]. Despite the support of PBT for improved disease control and decreased long-term risk, barriers to PBT access are often multifaceted, including clinical, systemic, financial, and age-related challenges. In the United States, there are 46 centers that offer PBT [18]. An initial barrier to PBT is geographic location, as residence of patients may limit access to institutions that offer PBT. Pursuit of PBT may require patients to travel long distances, which can lead to time away from family, work, and school. Additionally, patients and families may have to arrange for local accommodation during both treatment planning and delivery. These geographic and logistical challenges outline physical barriers to care, which may contribute to mental and financial stress, especially when faced with PBT denials and/or delays in treatment. While current research supports PBT for the treatment of the AYA population for disease control, reduced risk of recurrence, and reduced risk for long-term radiation-related toxicities, additional research should explore how social determinants of health influence the pursuit of PBT.

A cross-sectional cohort study investigated insurance approval and appeal outcomes for PBT among young adult patients compared to pediatric patients at a large-volume PBT center. This cross-sectional cohort study analyzed 284 consecutive patients aged 0–39 who were recommended PBT between 2018 through 2019. Tumor type and location, approval rates, denial rates, and decision time were evaluated as factors influencing insurance decisions [14]. Findings revealed that young adult patients were significantly less likely to receive approval for PBT compared to pediatric patients, regardless of tumor type or location [14]. Insurers justified denial decisions by citing lack of randomized data supporting PBT and its high cost. While PBT is an expensive treatment modality upfront, it may offer positive long-term cost-effectiveness, due to the ability to minimize long-term toxicities. Despite evidence outlining the benefit of PBT in the AYA cancer populationresults of the cross-sectional cohort studyrevealed that treatment approval rates were lower than expected.

In addition to lower approval rates, AYAs with cancer also experience more delays in obtaining insurance approval compared to pediatric patients. Prior authorization (PA) contributes to these delays, often requiring a physician to appeal the decision of denial. According to the Alliance for Proton Therapy [18], 42.8% of PA requests for PBT were initially denied. Furthermore, a national report by The Alliance for Proton Therapy Access released in 2018 highlighted the barriers to obtaining insurers’ approval for PBT. Data from several proton therapy centers revealed that nearly two-thirds (63%) of cancer patients aged 18–64 whose physicians recommended proton therapy as the best course of treatment were initially denied by their insurer [18]. Even when appeals reversed the first decision of denial, the appeal process resulted in significant waiting periods that led to treatment delays. PBT was found to be denied more than four times out of ten (42 percent), while averaging more than five weeks (27 working days) to receive the final denial [18]. These statistics outline the high rate of PBT denial, as well as treatment delays caused by the appeal process, which may still result in denial of treatment. A single institution retrospective analysis further solidified the challenges associated with PA for PBT. Treatment start times and denial rates were compared between patients considered for PBT. Of the 444 patients considered for PBT, The American Society for Radiation Oncology model policy supported coverage for 77% of the patients [19]. Of the adult patients requiring PA, 64% were initially denied and 32% remained denied after appeal [19]. Furthermore, no clinical characteristics predicted approval or denial and patients with commercial insurance were more likely to receive an initial denial [19]. Through this process, treatment initiation was delayed by an average of three weeks (up to four months) for adult patients requiring appeal, which then resulted in patients ultimately abandoning treatment. Both discussed studies outline the challenge of insurance approval for the AYA population in pursuing PBT. Denial of PBT and/or delays in the treatment can have an impact on disease control, as well as increase stress for the patient and family.

Rationality for denial has been attributed to lack of representation in clinical studies that may validate the need for PBT in the treatment of AYA sarcoma patients. A systematic review of barriers and facilitators to clinical trial enrollment among AYA patients with cancer was completed in 2020 by Siembida and colleagues. Of the 155 manuscripts examined, 13 manuscripts were used in the final analysis. The outlined barriers to enrollment in cancer clinical trials (CCT) included a lack of existing trials applicable to the patient population, limited access to available CCTs, and a lack of physician awareness of relevant trials [20]. The COG AYA Oncology Discipline Committee conducted a cross-sectional survey to identify barriers and facilitators to clinical trial enrollment. With a 42% response rate (60/143) and 97% survey completion, key barriers included administrative logistics (45%), inconsistent enrollment practices (42%), communication gaps between pediatric and medical oncology (27%), and limited trial availability (27%). Facilitators to enrollment included effective communication between pediatric and medical oncology(48%), supportive research infrastructure (35%), and the presence of AYA champions (33%) [21]. The results of the survey offer opportunities to address known barriers and implement facilitators to increase clinical trial enrollment for AYA patients so that there may be additional data to support PBT utilization. The barriers discussed provide compelling evidence for the need to reform insurance approval processes for AYA cancer patients, highlight geographic location as a limiting factor to access, and reinforce the critical importance of delivering timely care. Utilization of PBT and limiting delays in starting treatment promote exceptional patient care in the present and facilitate improved future outcomes due to the decreased toxicity risk.

### 3.3. Developmental and Psychosocial Considerations in AYA Care

#### 3.3.1. Developmental Milestones of an AYA Patient

A sarcoma diagnosis can occur at any point in an AYA’s life—during high school, just before college, while in college, at the start of a new job, between jobs, while planning a wedding, or when trying to start a family. Such a diagnosis can put important developmental milestones to a sudden halt. Many patients have been forced to step away from a hard-earned job, delay starting college alongside their peers, pausing their education, or rely more heavily on parental support. Ultimately, such a diagnosis can leave the lives of AYA patients feeling stagnant and disrupted.

A 2022 review examined the typical developmental milestones within the AYA population and highlighted the disruptive effects of cancer. The review subdivided the AYA group into three developmental categories: late adolescence (ages 15–18), emerging adulthood (ages 18–25), and young adulthood (ages 25–39) [22].

**Late adolescents (15–18)** are experiencing puberty and ongoing frontal brain development. Psychosocially, they are forming identities, exploring sexuality, and achieving key legal milestones such as driving, voting, and drinking [22].

**Emerging adults (18–25)** may be completing education, beginning careers, forming relationships, and considering starting families. By age 25, frontal brain development is typically complete.

**Young adults (25–39)** are often more established—professionally, financially, and within family life [22].

Despite developmental differences, individuals across all AYA age groups experience disruptions to physical and mental health, concerns about fertility and sexual health, and significant financial strain from diagnosis to survivorship.

#### 3.3.2. Fertility Risk and Preservation

**Figure 3:**
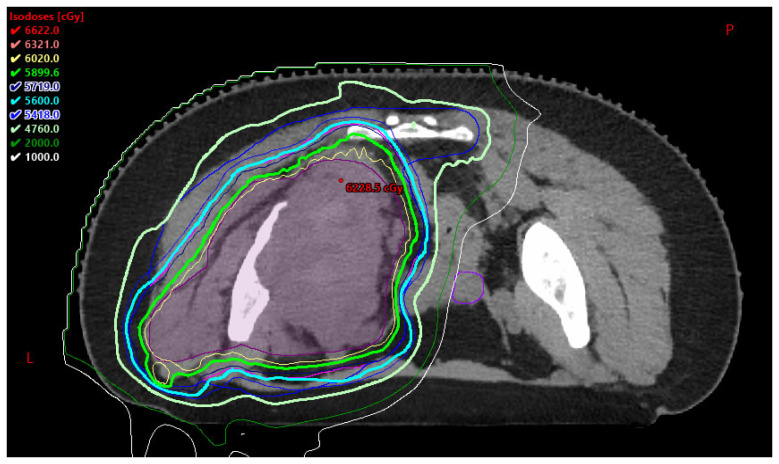
Representative axial image of a PBT plan for an AYA patient with unresectable pelvic Ewing sarcoma treated to 60.2 Gy in 28 fractions. PBT permitted a reduced dose to the contralateral ovary represented in purple. P: posterior L: left.

It can be hypothesized that PBT may assist in reducing long-term fertility risks by minimizing the dose to genitourinary organs, the pelvis, and the hypothalamic-pituitary axis [14]. This is highlighted in Figure 3., which is an axial image of a PBT plan for an AYA patient with unresectable pelvic Ewing sarcoma treated to 60.2 Gy in 28 fractions. PBT permitted a reduced dose to the contralateral ovary represented in purple. An animal study was conducted in 2021 comparing anti-Müllerian hormone (AMH) levels (a biomarker of ovarian function) and primordial follicle survival after in vivo mouse pelvic gamma-ray radiation therapy (GRT) versus PBT [23]. Findings suggest that PBT can preserve ovarian function when ovaries are positioned distal to the spread-out Bragg peak (SOBP) in tumors of the abdominopelvic region [23], thereby demonstrating PBT as a superior modality for protection of fertility organs and encouraging future research on use of PBT in protection of fertility organs in women.

Despite the potential of PBT to protect fertility organs, decrease long-term fertility risks, and general long-term toxicities, the risk of fertility impairments as a result of radiation therapy necessitates an early discussion regarding fertility preservation (FP) options among AYA cancer patients. The American Society of Clinical Oncology (ASCO) first published evidence-based clinical practice guidelines on FP in 2016, with the most recent update in 2025. This systematic review guideline aim was to provide guidance on assessing, discussing, and offering FP to patients with cancer [24]. The following recommendations were made:

- Cancer patients should be evaluated and counseled regarding reproductive risks at diagnosis and during survivorship.
- Patients interested in FP should be referred to a reproductive specialist.
- FP approaches should be discussed before therapy is initiated:
  ○ Males: sperm cryopreservation.
  ○ Females: embryo, oocyte, and ovarian tissue cryopreservation (OTC), ovarian transposition, and conservative gynecological surgery.
- For patients requiring urgent oncology therapy, GnRH may be offered for menstrual suppression.
- Advocate for comprehensive FP services coverage and support access to benefits [25].

Attitudes toward and practices of FP discussion among AYA patients with cancer were explored through semi-structured interviews with medical oncologists and young adults (YA). Among the themes identified, YAs emphasized the importance of having adequate knowledge to support informed decision making about fertility, with frustration expressed regarding the lack of information provided on the potential impact of treatment on fertility [25]. Costs associated with assisted reproduction were discussed, as well as the influence of cultural/religious beliefs on decision making. Themes identified among medical oncologists included clinical factors to determine recommendations surrounding fertility, the interest of patients in children/having more children, and the unknown risk to fertility when newer therapies are utilized in treatment of the patient’s cancer. These findings highlight the necessity of thorough education on treatments’ potential impact on fertility, starting at diagnosis, as well as information on FP.

In addition to education on FP, an AYA sarcoma patient may experience a financial burden due to costs associated with FP. The Alliance for Fertility Preservation outlines the spectrum of cost for preservation for men and women. Preservation options for men include sperm banking, testicular sperm extraction, and electroejaculation, while women’s options include egg freezing, embryo freezing, ovarian tissue freezing, ovarian transposition, or ovarian suppression [26]. Costs for fertility preservation for men range from $500 and $12,000, which does not include storage cost for certain services. Costs for fertility preservation for women can be anywhere between $10,000 and $15,000, before storage fees [26]. In addition to the cost of FP, those who may have difficulty with fertility because of cancer treatment may also experience a significant financial burden. The average cost of a single IVF cycle can be anywhere from $14,000 to $20,000 for a single cycle, not including cost of medications, genetic testing, cryopreservation, or storage fees [27]. Financial concern related to oncofertility was explored through a qualitative study in which dominant themes included varied access to health insurance, presence of parental/guardian support, reliance upon financial aid, negotiating infertility risk, and lack of preparation for long-term costs [28]. It was also found that many AYAs rely heavily upon parents for out-of-pocket costs and insurance coverage support [28]. The explored topics further highlight the benefit of PBT when caring for AYA sarcoma patients, which reinforces the need for conversations about the potential impact of cancer treatment on fertility and FP, as well as the financial impact of FP.

#### 3.3.3. Sexual Health

The impacts of cancer treatment on sexual health are important considerations for an AYA sarcoma patient. The WHO defines sexual health as “a state of physical, emotional, mental and social well-being related to sexuality; it is not merely the absence of disease, dysfunction or infirmity” [29]. A comprehensive review exploring survivorship considerations and management in the AYA sarcoma population, highlighted the limited research available on sexual health in AYA sarcoma patients and survivors [30]. Building on this review, the Adolescent and Young Adult Health Outcomes and Patient Experience (AYA HOPE) study, explored the impact of cancer on sexual function and intimate relationships. Of the 465 participants, a negative impact on sexual function was reported by 49% of participants one year after diagnosis and 43% two years after diagnosis [31]. Additionally, participants reporting fatigue and a negative perception of their physical appearance were more likely to report a negative impact on sexual function [31]. The findings about cancer treatment’s negative impact on sexual function were echoed in a longitudinal study, which examined the prevalence of sexual dysfunction among AYA cancer patients four months, six months, and two years after the initial diagnosis. More than half of the participants reported a problem with sexual function at each assessment, with most cases being female patients, married or in a committed relationship, treated with chemotherapy, psychological distress, and lower social support [32]. The above findings support the necessity of assessing sexual function and sexual health outcomes (SHO) throughout the continuum of care.

A systematic review and a meta-analysis were conducted, summarizing the recent literature on the impact of AYA cancer treatment on sexual health outcomes (SHO). Out of the articles obtained, eight were included that investigated 23 different SHO in 9038 AYA cancer patients. Outcomes explored the impact on desire, arousal, orgasm, and other in males, and desire, arousal, orgasm, pain, and other for females. Results revealed increased ejaculatory dysfunction and reduced testosterone levels in males and increased vaginal dryness in female AYA cancer patients [33]. Both clinical data and AYA childhood cancer survivors’ experiences with sexual dysfunction were explored. Themes of the semi-structured interviews included who, how, when, and where sexual dysfunction conversations should occur. AYA childhood cancer survivors stressed the importance of patient–provider rapport, normalizing conversations around sexual health, and desire for a screening tool to facilitate such conversations due to their personal nature [34]. While some research has examined sexual health among AYA childhood cancer survivors, there remains a clear need to explore sexual health among AYA cancer patients throughout the continuum of care. To further investigate the gap in identifying and addressing sexual health concerns of AYAs, point-of-care assessment of sexual concerns among active patients and survivors was examined. A total of 127 YAs (19–39) completed versions of the NCCN Distress Thermometer and Problem List (AYA-POST; AYA-SPOST), developed specifically for AYAs as part of an ongoing needs assessment study. At least one sexual health concern was reported by 27.6% of the total sample, with those who were actively undergoing treatment with sexual concerns reporting higher stress, while all genders reported general sexual concerns and loss of libido [35]. Given the limited research on sexual health among AYA cancer patients, there is a future opportunity to develop tools that support and facilitate the discussion around sexual health from diagnosis through to survivorship.

#### 3.3.4. Long-Term Financial Burden

While the cost of FP and challenges to sexual health have been discussed, the overall cost of care and financial impact on AYA patients must be explored. A report commissioned by Teen Cancer America and performed by Deloitte Access Economics in 2021 revealed that the economic and human cost of cancer in AYAs is substantial—$23.5 billion overall, with 259,234 per person over their lifetime [36]. According to the Mayo Clinic’s online cost estimator, the cost of a single PBT treatment was approximately $5904 at the end of 2020 [37]. Given that AYA sarcoma patients typically undergo five weeks of daily radiation treatment, the total cost of PBT amounts to approximately $147,600. These expenses, not always fully covered by insurance, may contribute to the financial hardship experiences among patients and survivors.

A systematic review explored financial hardships experienced by cancer survivors. Financial hardship was categorized by material conditions (e.g., out-of-pocket costs, productivity loss, medical debt, or bankruptcy), psychological responses (e.g., distress or worry), and coping behaviors (e.g., skipped medications) [38]. The majority of the studies examined found financial hardship to be a material condition measure, with 12–62% of survivors reporting being in debt due to the cost of treatment, 47–49% reporting some form of financial distress, and 4–45% of survivors reporting lack of adherence to medication due to cost [38]. While the previous review explored financial hardships among all cancer survivors, the impact of financial burden on an AYA sarcoma cancer patient must be explored, due to heightened risk for long-term financial hardship.

AYA survivors were surveyed to explore the impact of discussions on financial burden and relationships on decreased long-term financial toxicity in AYA cancer survivors. Of the surveyed AYA survivors, 70% reported not recalling any conversations with a provider about the cost of treatment—communication about potential financial burden was found to be associated with decreased financial toxicity [39]. Survivors also expressed frustration over lack of discussions regarding financial hardship from the time of diagnosis treatment through to survivorship. It is important to acknowledge that AYA sarcoma cancer patients may experience direct and indirect costs related to their cancer care. As previously highlighted, a direct cost related to cancer care may be FP. FP is costly, typically not covered by insurance, and does not include storage fees. Indirect costs that could be explored may be loss of income due to time away from work, prolonged education due to time away for treatment, and transportation/lodging for specialized care (especially in the case of PBT).

The literature has provided a rationale for an AYA patient’s heightened risk for financial burden and toxicity; YAs are the age group at greatest risk for unemployment, with 12.4% of YAs aged 20–24 years and 7.4% of those aged 25–34 years being unemployed [40]. Adolescents/young adults are also the most likely patient population to not have insurance. To further explore the financial burden of cancer in young adults, a retrospective analysis was performed on data collected from the Samfund grant applications of 334 YA cancer survivors. The data suggested that the financial impact of cancer is more severe in young adults aged 30–39 compared to those under 30 [40], with the older young adult population more likely to have less or no parental support, with a greater chance of having dependents/finances related to home ownership. Medical debt in the sample studied rose sharply in survivors aged 26–30 years in comparison to younger young adults aged 22–25 years: a 23.8% increase [40]. Long-term financial experiences were highlighted through a cross-sectional, anonymous survey of AYA cancer survivors. Financial hardship and high financial stress were observed, with the following financial consequences reported: post-cancer credit score decrease (44%), debt collection contract (39%), spending more than 10% of income on medical expenses (39%), and lacking money for basic necessities (23%). Financial coping behaviors included taking money from savings (55%), taking on credit card debt (45%), putting off major purchases (45%), and borrowing money (42%) [41]. Financial hardship may also result in medical nonadherence, resulting in overall poorer outcomes [40]. Awareness and acknowledgment of the financial impact of cancer on adolescent and young adult (AYA) sarcoma patients is essential to evaluating and enhancing supportive care. Improved supportive care that addresses financial burden has been associated with better physical and mental health outcomes among AYA survivors [40]. Future research should explore how financial hardship affects key aspects of survivorship, including loss of follow-up, adherence to surveillance imaging, and continuation of necessary medications.

#### 3.3.5. Palliative Care and Goals of Care

Addressing the financial hardships faced is just one aspect of delivering comprehensive care to AYA cancer patients. Utilization and integration of palliative care at the time of diagnosis is essential for establishing goals of care and honoring values, and supporting symptom management during and after treatment, while also facilitating a holistic approach to care. The WHO defines palliative care as an approach that improves the quality of life of patients—adults and children—and their families who are facing problems associated with life-threatening illness, which assists patients by preventing and relieving suffering through early identification, impeccable assessment, and treatment of pain and other problems, whether physical, psychosocial, or spiritual [42]. Nearly 90% of AYAs with cancer reported distressing symptoms during their treatment; those who survived reported ongoing unmet psychosocial and physical health needs, and those who died from their disease were highlighted to have likely received medically intensive care that was not aligned with their goals and values [43]. Integration of palliative care is an exceptional and necessary tool to support an AYA cancer patient as a whole person.

A retrospective cohort study reviewed social determinants of health that have impacted the experience of young adults with cancer at a single community urban hospital, which revealed that palliative care was underutilized (44/175; 25%) and frequently consulted when the patient was near the end of life (70%) [44]. Underutilization of palliative care was also demonstrated in a recent cross-sectional study in California that reviewed AYA cancer patients who were 12 to 39 years of age at death. The aim of the study was to explore frequency, timing, and evolution of AYA patients with cancer in the last 90 days of life. Of the 1929 AYA patients with cancer, documented palliative goals of care (GOC) increased as patients approached death, from 7.2% of patients 61 to 90 days before death to 17.2% 31 to 60 days before death, and 57.5% in the last 30 days, with 20.4% of patients transitioning from non-palliative to palliative goals before death [45]. Results also highlighted that non-white patients were disproportionately represented among those not having documented GOC discussions. The exploration of the timing of palliative care integration and impact on end-of-life outcomes revealed that earlier referrals were associated with fewer intensive measures near death, intensive care unit admissions, emergency room visits, and hospitalizations in the last month of life [46]. Furthermore, early referrals also enhanced attention to symptoms experienced in the last 90 days of life, such as pain, dyspnea, nausea, diarrhea, constipation, depression, and anxiety [46]. The research discussed demonstrates the necessity of inclusion of palliative care earlier on for AYA cancer patients, so that goals of care and patient wishes are clearly defined, as well as additional research on the disproportions of GOC among diverse populations. Future research opportunities may include the development of a needs assessment tool to guide the timing of referrals for AYA cancer patients to palliative care—whether that be for support during treatment through transition to survivorship, or for a single visit with the aim of discussing the goals of care.

#### 3.3.6. Survivorship

Based on estimates of new cancer cases in 2025, 4.2% of all new cases will occur among AYAs, and of those diagnosed with cancer, 86% will survive their cancer for five years after diagnosis [17]. Addressing health-related quality of life is an important component of cancer survivorship. Health-related conditions among AYA long-term cancer survivors were explored through utilization of the SURVAYA questionnaire study. Of the 3776 AYA survivors, 58.5% experienced health-related conditions after their cancer diagnosis, of whom 51.4% were diagnosed with two or more conditions. Reported conditions included vision (15.0%); digestive system (15.0%); endocrine system (14.1%); cardiovascular system (11.7%); respiratory system (11.3%); urinary tract system (10.9%); depression (8.6%); hearing (7.4%); arthrosis (6.9%); secondary malignancy (6.4%); speech, taste, and smell (4.5%); and rheumatoid arthritis (2.1%) [47]. The high prevalence of health-related conditions among AYA cancer survivors highlights a need for individualized survivorship care and further exploration of how cancer care impacts health-related quality of life. The SURVAYA questionnaire study was also utilized to compare HRQOL in AYA cancer survivors versus a normative population. AYA cancer survivors were found to experience worse HRQOL compared to peers in all explored domains—cognitive functioning, role functioning, social functioning, physical functioning, and emotional function, with cognitive functioning being predominately worse [48].

In addition to impacts on HRQOL, long-term radiation toxicities must also be explored. A cross-sectional study explored AYAs with cancer who received (radiation therapy) RT, to identify RT-related toxicities and examine their impact on health-related quality of life (HRQOL). While there are known benefits to PBT for disease control, cure, and palliation, patients are still at risk for acute and long-term radiation-related toxicities. Of the 84 AYA patients that completed the surveys during RT, 89% experienced acute radiation therapy (RT) side effects, which were primarily grade 1 (65%), but those who experienced acute grade 2 or greater reported worse mental health and pain [49]. The post-RT survey (14–27 months after treatment) similarly found late RT-related toxicities to be grade 1 (51%) [50]. However, those that experienced late grade 2 toxicities or more reported worse mental health, worse social roles, and greater sleep disturbance [50]. The presence of acute or late RT grade 2 or greater toxicities was found to contribute to a worse health-related quality of life, supporting the notion that early detection and management of side effects throughout the course of treatment is needed.

The needs of AYA cancer survivors should also be explored. A concept-mapping analysis was completed by in order to better understand the survivorship needs of AYA cancer survivors [51]. AYA cancer survivors identified the following as priority components of survivorship: factors impeding life goals, positive life changes after treatment, development of a new identity, financial toxicity as a result of treatment, and fears after cancer treatment. Navigating relationships, psychosocial difficulties, cognitive effects of treatment, negative psychosocial effects, and physical effects of treatment were identified as the areas least addressed in the transition to survivorship. Finally, AYA survivors felt the least prepared for post-treatment support, ongoing emotional effects of treatment, cognitive effects of treatment, negative psychosocial effects of treatment, and physical effects of treatment [51]. Findings indicate the many components of survivorship for an AYA cancer patient, as well as the opportunity to better support AYA cancer patients as they transition to survivorship.

### 3.4. Future Directions for the Support of AYA Sarcoma Patients

#### 3.4.1. Role of Social Media and Cancer Care Information

The AYA patient population has been found to have high rates of social media usage, with up to 90% of adults aged 18–29 using social networking sites [49]. Social media platforms have been explored as tools of support for AYA patients with sarcoma, both for acquiring health information and connecting with fellow survivors [52]. A 2021 study by Donnovan et al. explored the role of social media in providing support from friends for AYA patients and survivors of sarcoma through conducting semi-structured interviews. Themes from the study highlighted the advantages and disadvantages of social media for an AYA cancer patient. Study participants expressed desire for connection with friends, feelings of no longer fitting in, and the difficulty in seeing those close to them “move on” with their lives. Social media was also identified as a tool to connect with other AYA patients, bringing about a sense of connection and understanding. Connecting with friends and other AYA patients with sarcoma through social media was found to be important in providing support for AYAs with sarcoma but also demonstrated the potential to amplify feelings of frustration and anxiety [53]. This qualitative study revealed the positive and negative aspects of social media in relation to AYA cancer patients. Continued exploration of social media use and impact on the AYA cancer patient may deem social media beneficial for future development of safe and supportive platforms that can be utilized from diagnosis and into survivorship.Beyond fostering connection, social media may also serve as a tool for education on cancer care. Age differences in the patterns of and confidence in using the internet and social media for cancer care among cancer survivors was explored through a cross-sectional survey that evaluated cancer-related internet and social media use, while also exploring confidence in utilizing these resources.A total of 138/371 cancer survivors were AYA and were more likely to use social media for cancer care [54]. Topics important to AYA patients (fertility information, sexual health, and body image information) were commonly researched, as well as information on physician ratings, physical activity information, and support groups [54]. Additionally, common information explored by the AYA cancer survivor included information on diet and nutrition, online cancer specific communities, fertility information, body image, sexual health information, face-to-face support groups, and return to school or work information [54]. Results revealed that the most common websites utilized for cancer-care-related information included cancer society websites (59%), WebMD (48%) and the local institutional website (31%), whereas the most common social media platforms were Facebook (37%), YouTube (14%), and Instagram (9%) [54]. Awareness of social media use, commonly searched subjects, and preferred social media platforms may assist in the use of social media for AYA cancer care education from diagnosis to survivorship.

A recent review explored the role of social media in the provision of high-quality care within the AYA oncology context. Results of the review were categorized into social media as a lifeline, promises of integrating social media into AYA oncology, barriers, and recommendations. Social media was highlighted as a “lifeline” to the outside world, as well as a source of information. Interestingly, the desire to access cancer information was found to be dependent on where the patient was in their cancer timeline [55]. When undergoing treatment, an AYA patient was found to prefer information on cancer management to come from their treatment team. At this point in time, social media was found to be used for entertainment and staying in touch with family and friends [55]. Results of this highlight the opportunity for AYA cancer content to be integrated into educational resources for AYA patients.

Another area of exploration was the use of social media as a driver of behavioral changes. The reviewed articles highlighted the positive impact of social media use for patients, such as a smartphone medication reminder, online cookbooks for health recipes, physical activity programs, and computer-mediated support groups to combat cancer-related fatigue, as well as a health-related quality of life (QOL) tool for retinoblastoma survivors, which allows the medical team to initiate a discussion about psychosocial difficulties [55]. Supportive social media tools for an AYA cancer patient from diagnosis to survivorship should be further explored. Social media tools were found to be useful for educational purposes, as well as to improve psychosocial care.

The lower and upper age of an AYA cancer patient poses a range of differences when it comes to life experiences and developmental stages, further highlighting the uniqueness of this cancer patient population. The reviewed literature found that psychosocial support and care is underutilized due to practical challenges, such as transportation to supportive services, severity of illness and inability to travel, lack of offering of supportive services, and stigma around mental health [55]. This information calls upon cancer care facilities to examine present supportive services for patients and how that could be tailored to support the needs of an AYA cancer patient. An additional barrier discussed was the lack of accessibility of AYA-specific information. The limitations highlighted included difficulty in finding AYA quality sources, difficulty in understanding information due to medical jargon, and excess text in content [55]. With the rise of TikTok and continued use of Instagram, Snapchat, and Facebook, additional research could explore these platforms and how they can be better utilized in the development of tailored content for the AYA cancer patient. The explored literature exemplifies the potential of social media integration to improve the experience of an AYA cancer patient throughout the continuum of care.

#### 3.4.2. Development of an AYA-Specific Program and Future Opportunities

In 2008, an AYA program was started at CancerCare Manitoba (CCMB). A commentary piece was completed that outlined how the program was established and the factors that influenced its success. Information was provided with the hope that its program may facilitate influence for the implementation of AYA programs to improve the care and outcomes of the AYA population. CCMB receives an average of 300 referrals per year of newly diagnosed AYAs with cancer, highlighting the necessity of AYA-specific supportive programs. According to Scott and Oberoi, long-term childhood cancer survivors are often the forgotten demographic within AYAs with cancer, so it is important to also recognize this group of AYS when it comes to program planning and development. The AYA program in Manitoba led focus groups in 2021 to better explore the unmet needs of the AYA patient population. Common themes included reducing the delay at diagnosis, provision of AYA-specific information and supportive care resources, increased supportive care programs that address AYA-specific needs, improvement in access to clinical trials, and a smoother transition from treatment to survivorship [56]. While the results of the focus groups have not been published, the themes discussed are important to increase awareness and understanding and provide opportunities for improvement in the care of an AYA patient.

Due to the continued rise in AYA cancer patients, as well as time spent in survivorship, opportunities for changes to the model of care must be explored. In addition to assisting patients in expert facilities, the above topics explored also highlight opportunities for changes to be made in daily oncology clinic practice. While it is known that healthcare facilities have varying resources accessible, sometimes limited, an order set could be developed to support an AYA sarcoma patient. This may include early PT/OT referral, palliative care/discussion on GOC, social worker referral/directing patients to known external AYA cancer support groups, women’s health for discussion of sexual health/fertility health, urology for sexual health for men, dietician for general nutrition and well-being, and provision of services to support finances (grants, scholarships, etc.). Utilization of a needs assessment may be deemed beneficial, to ensure an AYA sarcoma patient’s needs are individually addressed.

Utilization of a needs assessment to address the needs of AYA patients during cancer treatment was explored through the conduction of a mixed methods single-arm feasibility pilot study [57]. The AYA Needs Assessment and Service Bridge (NA-SB) was developed, which included a needs assessment capturing AYA’s physical, psychosocial, and practical needs, as well as integrated referral pathways that are triggered depending on reported needs. Results demonstrated that the 26 AYA participants accepted the needs assessment and found it appropriate, and 77% of participants felt that their needs were met in the study period [57]. This pilot study provides evidence of the benefits of using a needs assessment to appropriately meet the needs of an AYA cancer patient. Future studies could explore the use of a needs assessment upon completion of cancer treatment and during survivorship.

## 4. Conclusions

**Figure 4:**
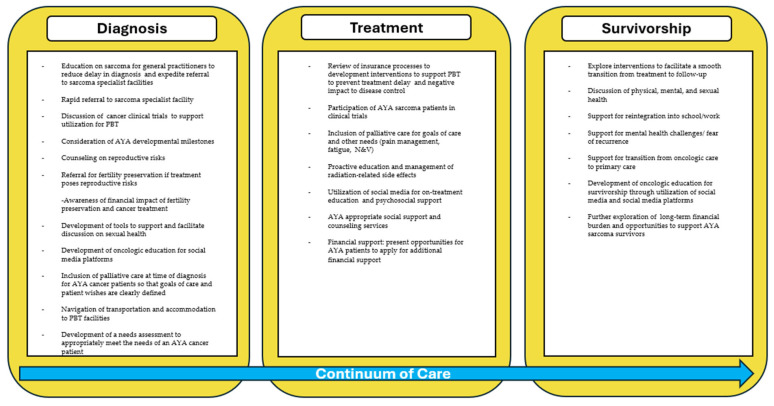
Summary of recommendations at the time of diagnosis, during treatment, and the transition to survivorship to best support an AYA sarcoma cancer patient.

This review highlights the importance of recognizing the unique challenges faced by adolescent and young adult (AYA) patients across the cancer care continuum—from the onset of symptoms, through diagnosis and treatment, and into survivorship (Figure 4).

It is essential for radiation oncology teams to have an understanding of this. Acknowledging these challenges can help identify gaps in care and uncover opportunities to better support this distinct population, ultimately leading to improved outcomes. The known oncologic side effects, particularly those associated with radiation therapy, underscore the value of PBT as a treatment option. Its precise targeting may reduce long-term toxicity, which is especially critical for AYAs. Therefore, increased advocacy for proton therapy is essential—not only in clinical decision making but also in addressing the barriers that hinder its approval and accessibility.

## Abbreviations

The following abbreviations are used in this manuscript:

AYA	Adolescent Young Adult
PBT	Proton beam therapy
WHO	World Health Organization
NCCN	National Comprehensive Cancer Network
CCC	Comprehensive Cancer Center
SPAGN	Sarcoma Patient Advocacy Global Network
CCT	Cancer clinical trials
IMRT	Intensity modulated radiation therapy
ASCO	American Society of Clinical Oncology
FP	Fertility Preservation
AMH	Anti-Müllerian hormone
GRT	Gamma-ray radiation therapy
SOBOP	Spread-out Bragg peak
YA	Young adults
SHO	Sexual health outcomes
GOC	Goals of care
RT	Radiation therapy
QOL	Quality of life
CCMB	CancerCare Manitoba
CPG	Clinical Practice Guidelines
MTB	Multidisciplinary tumor boards
NA-SB	Needs Assessment and Service Bridge
